# Supplementary material for: Dietary Biodiversity and Diet Quality in Dutch Adults
Source: Nutrients. 2024 Jul 9;16(14):2189. doi: 10.3390/nu16142189 (PMC11279674; doi:10.3390/nu16142189)
Supplement: Supplementary file 1 [file nutrients-16-02189-s001.zip › nutrients-3069346-supplementary.pdf]

Supplementary Materials

Dietary biodiversity and diet quality in Dutch adults.

Nutrients

Rosalie E. Bakker[1], Vera S. Booij[1], Corné van Dooren[2], Mary Nicolaou[3], Ingeborg A. Brouwer[1], Margreet R. Olthof[1]
[1]Vrije Universiteit Amsterdam, Department of Health Sciences, Faculty of Science, Amsterdam Public Health Research Institute, 1081 HV Amsterdam, The Netherlands
[2]WWF-NL, 3708 JB Zeist, The Netherlands.
[3]Amsterdam UMC,University of Amsterdam, Department of Public and Occupational Health, 1007 MB Amsterdam, The Netherlands.

Corresponding author:
Rosalie E. Bakker
E-mail address: rosalie.bakker@vu.nl

Contents Supplementary materials:

**Table S1**	Overview of species determined in the Dutch National Food Consumption Survey 2012-2016 respondents (adults 19-79 years).
**Table S2**	Food items and/or food groups included in the different Dietary Species Richness (DSR) scores (overall, fruit and vegetables) in the Dutch National Food Consumption Survey 2012-2016 respondents (adults 19-79 years).
**Table S3**	Components of the Dutch Healthy Diet Index 2015 (DHD15-index) and Dutch dietary guidelines including the threshold (minimum score) and cut-off (maximum score) values according to Looman et al. (2017) [1]
**Table S4**	The association between Dietary Species Richness (DSR) and diet quality (DHD15-index score) in the Dutch National Food Consumption Survey 2012-2016 respondents (adults 19-79 years) stratified by sex.
**Table S5**	Sensitivity analysis of the association between Dietary Species Richness (DSR) and diet quality (DHD15-index score) in the Dutch National Food Consumption Survey 2012-2016 respondents (adults 19-79 years) adjusted for BMI instead of total kilocalories intake.
**Figure S1**	Graph of different Dietary Species Richness (DSR) scores and the quantity consumed in grams per DSR score in the Dutch National Food Consumption Survey 2012-2016 respondents (adults 19-79 years).

Table S1. Overview of species determined in the Dutch National Food Consumption Survey 2012-2016 respondents (adults 19-79 years).

**Dutch common name**	**English common name**	***Species name***
Fruit		
Aardbei	Strawberry	*Fragaria x ananassa Duchene ex Rozier*
Abrikoos	Apricots	*Armeniaca vulgaris*
Ananas	Pineapple	*Ananas comosus*
Appel	Apple	*Malus domestica*
Banaan (excl. Bakbanaan)	Banana (excl. Plantain)	*Musa acuminata*
Blauwe bes	Blueberries	*Vaccinium corymbosum*
bosbes	Blueberries	*Vaccinium myrtillus*
Bramen	Blackberries	*Rubus fruticosus*
Citroen	Lemon	*Citrus limon*
Cranberry	Cranberries	*Vaccinium macrocarpon*
Dadel	Date	*Phoenix dactylifera*
Druif	Grapes	*Vitis vinifera*
Frambozen	Raspberries	*Rubus idaeus*
Goji bes	Goji berry	*Lycium barbarum*
Granaatappel	Pomegranate	*Punica granatum*
Grapefruit	Grapefruits	*Citrus paradisi*
Kaki	Kaki	*Diospyros kaki*
Kersen	Cherries (sweet and sour)	*Prunus avium*
Kiwi	Kiwi	*Actinidia chinensis*
Kokosnoot, seroendeng	Coconut	*Cocos nucifera*
Kruisbes	Gooseberry	*Ribes uva-crispa*
Limoen	Lime	*Citrus aurantifolia*
Lychee	Lychee	*Litchi chinensis*
Mandarijn	Mandarins	*Citrus reticulata*
Mango	Mango	*Mangifera indica*
Meloen (Galia, net, honing, suiker kanteloep	Melons	*Cucumis melo*
Moerbeien	Mulberries	*Morus (alba and nigra)*
Nectarine	Nectarines	*Persica vulgaris*
Olijf	Olives	*Olea europaea*
Papaja	Papayas	*Carica papaya*
Passievrucht	Passionfruit	*Passiflora edulis*
Peer	Pear	*Pyrus communis*
Perzik	Common peaches	*Prunus persica*
Pruim	Plum	*Prunus domestica*
Rode bes (aalbes)	Red currant	*Ribes rubrum*
Sinaasappel	Oranges	*Citrus sinensis*
Vijg	Fig	*Ficus Carica*
Watermeloen	Watermelon	*Citrullus lamatus*
Zwarte bes	Black currant	*Ribes nigrum*
Vegetables		
Rabarber	Rhubarb	*Rheum Rhabarbarum*
Alfalfa	Alfalfa	*Medicago sativa*
Andijvie, Groenlof	Endives	*Cichorium endivia*

Artisjok	Globe artichoke	*Cynara cardunculus*
Asperges	Asparagus	*Asparagus Officinalis*
Avocado	Avocado	*Persea americana*
Aubergine	Aubergine/eggplant	*Solanum melongena*
Komkommer, augurk	Cucumber, gherkin	*Cucumis sativus*
Bamboespruiten	Bamboo	*Bambusa vulgaris*
Bakbanaan	Plantain	*Musa × paradisiaca*
Biet	Beets, chards	*Beta vulgaris*
Selderij (bleek)	Celeriac, celery	*Apium graveolens*
Bonen	Common beans	*Phaseolus Vulgaris*
Champignons	Cultivated mushroom	*Agaricus bisporus*
Chantarellen	Chanterelles	*Cantharellus cibarius*
Courgette, pompoen	Courgette, pompoen, squash	*Cucurbita Pepo*
Erwten	Peas	*Pisum Sativum*
Kousenband	Asparagus bean	*Vigna unguiculata*
Kolen	Head cabbages	*Brassica Oleracea*
Chinese kool, paksoi, raapstelen	Chinese cabbages, turnip	*Brassica Rapa*
Sopropo	Bitter melon/goya	*Momordica charantia*
Spaanse peper	Chili peppers	*Capsicum frutescens*
Koolraap	Swede	*Brassica napus*
Schorseneren	Black salsify	*Scorzonera hispanica*
Kappertjes	Caper	*Capparis spinosa*
Knoflook	Garlic	*Allium sativum,*
Paardenbloem	Dandelion	*Taraxacum officinale*
Paprika	Sweet bellpeppers	*Capscum annuum*
Pastinaak	Parsnip	*Pastinaca sativa*
Postelein	Common purslane	*Portulaca oleracea*
Prei	Leek	*Allium ampeloprasum*
Sla	Head lettuces	*Lactuca Sativa*
Mais	Maize	*Zea Mays*
Spinazie	*Spinach*	*Spinacia oleracea*
Tauge	*Bean sprouts*	*Phaseolus aureus*
Tuinbonen	Broad beans	*Vicia Faba*
Tomaat	Tomato	*Solanum lycopersicum en Lycopersicon esculentum*
Ui	Onions	*Allium Cepa*
Venkel	Fennel	*Foeniculum vulgare*
Witlof	Belgian Endives	*Cichorium intybus*
Radijs	Radish	*Raphanus sativus*
Waterkers	Rorippa	*Rorippa nasturtium-aquaticum*
Wortel	Carrots	*Daucus carota*
Zeekraal	Salicornia	*Salicornia (not included in DSR vegetables)*
Zeewier (nori)	Nori	*Porphyra umbilicalis (not included in DSR vegetables)*

Zeewier Kelp	Kelp	*Saccharina japonica (not included in DSR vegetables)*
Tubers		
Aardappel (aardpeer)	Patato	*Solanum tuberosum*
Bataat	Sweet patato	*Ipomoea batatas*
Cassave, Tapioca	Cassava	*Manihot esculenta*
Pomtajer	Tannia	*Xanthosoma sagittifolium*
Yam	Yam	*Dioscorea alata*
Legumes		
Sojaboon	Soybean	*Glycine max*
Bonen (black-eyed)	Beans black eyed	*Vigna radiata*
Mungboon	Mung beans	*vigna radiata*
Kikkererwt	Chickpea	*Cicer arietinum*
Linzen	Lentil	*Lens culinaris*
Grains		
Haver	Oat	*Avena Sativa*
Boekweit	Buckwheat	*Fagopyrum Esculentum*
Gerst	Barley	*Hordeum Vulgare*
Gierst	Millet (pearl and finger)	*Panicum hirticaule*
Rijst	Asian Rice	*Oryza sativa*
Quinoa	Quinoa	*Chenopodium quinoa*
Rogge	Rye	*Secale cereale*
Tarwe	Wheat (common, spelt, durum, combinations)	*Triticum aestivum*
Nuts and seeds		
Amandelen	Almonds	*Amygdalus communis*
Cashew noot	Cashew	*Anacardium occidentale*
Pinda	Peanuts	*Arachis hypogaea*
Paranoot	Brazil nuts	*Bertholletia Excelsa*
Hennepzaad	Hemp seeds	*Cannabis Sativa*
Pecan noot	Pecan nuts	*Carya Illinoinensis*
Kastanje	Chestnuts	*Castanea spp.*
Hazelnoot	Hazelnuts	*Corylus avellana*
Walnoot	Walnuts	*Juglans regia*
Macadamia	Macadamia	*Macadamia integrifolia*
Pistachios	Pistachios	*Pistacia vera*
Zonnebloempitten	Sunflower seeds	*Helianthus annuus*
Lijnzaad	Line seeds	*Linum usitatissimum*
Maanzaad	Poppy seeds	*Papaver somniferum*
Pijnboompitten	Pine nuts	*Pinus spp.*
Chia zaad	Chia seeds	*Salvia hispanica*
Sesame zaad	Sesame seeds	*Sesamum indicum*
Animal-based species		
Koe	Cow	*Bos taurus*
Varken	Pig, boar	*Sus scrofa*
Geit	Goat	*Capra aegagrus*
Kalkoen	Turkey	*Meleagris gallopavo*
Schaap	Sheep	*Ovis aries*
Kip	Chcken	*Gallus gallus*
Eend	Duck	*Anas platyrhynchos*

Fazant	Pheasant	*Phasianus colchicus*
Struisvogel	Ostrich	*Struthio camelus*
Paard	Horse	*Equus ferus*
Konijn	Rabbit	*Oryctolagus cuniculus*
Hert	Red deer	*Cervus elaphus*
Ansjovis	Anchovies	*Engraulis encrasicolus*
Baars	European perch	*Perca fluviatilis*
Koolvis	Coalfish	*Pollachius virens*
Kabeljauw	Cod	*Gadus callarias*
Schar	Common dab	*Limanda limanda*
Paling	European eel	*Anguilla anguilla*
Krab	Edible crab	*Cancer pagurus*
Haring	Herrings	*Clupea harengus*
Heilbot	Halibut	*Hippoglossus hippoglossus*
Kreeft	American lobster	*Homarus americanus*
Schelvis	Haddock	*Melanogrammus aeglefinus*
Makreel	Atlantic Mackerel	*Scomber scombrus*
Mosselen	Common mussel	*Mytilus edulis*
Pangasius	Pangasius	*Pangasius pangasius*
Schol	European plaice	*Pleuronectes platessa*
Gamba	Northern prawn	*Pandalus borealis*
Forel	Trout (rainbow)	*Salmo gairdneri*
Zalm	Atlantic salmon	*Salmo salar*
Sardines	European sardines	*Sardina pilchardus Walbaum*
Garnaal	Common shrimp	*Crangon crangon*
Tong	Common sole	*Solea solea*
Inktvis	Squids	*Loligo forbesi*
Wijting	*Merlangius*	*Merlangius merlangus*
Tilapia	Nile tilapia	*Oreochromis niloticus*
Tonijn	Tuna	*Thunnus alalunga*

Table S2. Food items and/or food groups included in the different Dietary Species Richness (DSR) scores (overall, fruit and vegetables) in the Dutch National Food Consumption Survey 2012-2016 respondents (adults 19-79 years).

	**Overall DSR**	**DSR Fruit**	**DSR Vegetables**
Included	Cut-up, packaged, tinned, jarred and/or (deep)frozen foods.	Cut-up, packaged, tinned, jarred, (deep)frozen and unprocessed fruits.	Cut-up, packaged, tinned, jarred, (deep)frozen and unprocessed vegetables.
Included food groups	Fruits, vegetables, nuts and seeds, legumes, tubers (including potatoes), grains, meat, fish and dairy.	Fruits.	Vegetables incl. avocado, plantain, several varieties of beans and several varieties of peas.
Other foods	Tofu, Tahoe and Tempeh as meat substitutes (soy bean)		
Excluded	(Non) Alcoholic beverages (incl. coffee & tea).	Further processed fruit or fruit products.	Further processed vegetables or vegetable products.
	Savory snacks, sugar, chocolate, confectionery.		Salicornia, Nori and Kelp
	Oils & fats.		
	Condiments, spices & sauces.		
	Further processed meat substitutes.		

Table S3. Components of the Dutch Healthy Diet Index 2015 (DHD15-index) and Dutch dietary guidelines including the threshold (minimum score) and cut-off (maximum score) values [1].

	Component	*Advised consumption according to Dutch dietary guidelines 2015*	*Minimum score (= 0 points)*	*Maximum scores (= 10 points)*
**1**	Vegetables	Eat at least 200 g of vegetables daily	0 g/d	≥200 g/d
**2**	Fruit	Eat at least 200 g of fruit daily	0 g/d	≥200 g/d
**3**	Wholegrain products*	a. Eat at least 90 g of wholegrain products daily<br>b. Replace refined cereal products by wholegrain products	a. 0 g/d<br>b. No consumption of wholegrain products<br>OR<br>Ratio of whole grains to refined grains ≤0.7	a. ≥90 g/d<br>b. No consumption of refined products<br>OR<br>Ratio of whole grains to refined grains ≥ 11
**4**	Legumes	Eat legumes weekly	0 g/d	≥10 g/d
**5**	Nuts	Eat at least 15 g unsalted nuts daily	0 g/d	≥15 g/d
**6**	Dairy **	Eat a few portions of dairy produce daily, including milk or yoghurt.	0 g/d OR ≥ 750 g/d	300–450 g/d
**7**	Fish ***	Eat one serving of fish weekly, preferably oily fish	0 g/d	≥ 15 g/d
**8**	Tea	Drink three cups of black or green tea daily	0 g/d	≥ 450 g/d
**9**	Fats and oils	Replace butter, hard margarines, and cooking fats by soft margarines, liquid cooking fats, and vegetable oils	No consumption of soft margarines, liquid cooking fats and vegetable oils<br>OR<br>Ratio of liquid cooking fats to solid cooking fats <0.6	No consumption of butter, hard margarines and cooking fats<br>OR<br>Ratio of liquid cooking fats to solid cooking fats ≥ 13
**10**	Red meat	Limit consumption of red meat	≥ 100 g/d	≤ 45 g/d
**11**	Processed meat	Limit consumption of processed meat	≥ 50 g/d	0 g/d
**12**	Sweetened beverages and fruit juices	Limit consumption of sweetened beverages and fruits juices	≥ 250 g/d	0 g/d
**13**	Alcohol	If alcohol is consumed at all, intake should be limited to one Dutch unit (10 g ethanol) daily	Women:<br>≥ 20 g ethanol/d<br>Men:<br>≥ 30 g ethanol/d	Women:<br>≤10 g ethanol/d<br>Men:<br>≤ 10 g ethanol/d
**14**	Salt	Limit consumption of table salt to 6 g daily	≥ 3.8 g Na/d	≤ 1.9 g Na/d

g/d = gram per day, Na/d = Natrium per day
* This component comprises two sub-components (a and b). Each sub-component has a maximum score of 5 points.
** Maximum of 40 g cheese can be included.
*** Maximum of 4 g lean fish can be included.

Table S4. The association between Dietary Species Richness (DSR) and diet quality (DHD15-index score) in the Dutch National Food Consumption Survey 2012-2016 respondents (adults 19-79 years) stratified by sex.

**DSR Overall°**	**Men N=1043**	**Women N=1035**
Model 1[1] *B [95%-CI], p-value*	1.04 [0.81-1.27] <0.001*	1.50 [1.28-1.71] <0.001*
Model 2[2] *B [95%-CI], p-value*	1.22 [1.00-1.43] <0.001*	1.57 [1.36-1.78] <0.001*

**DSR Fruit**	**Men N=1043**	**Women N=1035**
Model 1[1] *B [95%-CI], p-value*	4.04 [3.46-4.63], <0.001*	3.89 [3.36-4.42], <0.001*
Model 2[2] *B [95%-CI], p-value*	4.08 [3.54-4.62] <0.001*	3.97 [3.46-4.48] <0.001*
Model 3[3] *B [95%-CI], p-value*	1.73 [0.99-2.47] <0.001*	2.06 [1.36-2.76] <0.001*

**DSR Vegetables**	**Men N=1043**	**Women N=1035**
Model 1[1] *B [95%-CI], p-value*	0.34 [-0.01-0.69], 0.06	1.24 [0.87-1.60], <0.001*
Model 2[2] *B [95%-CI], p-value*	0.46 [0.13-0.79], 0.006*	1.23 [0.89-1.57], <0.001*
Model 3[3] *B [95%-CI], p-value*	0.02 [-0.30-0.34] 0.90	0.27 [-0.07-0.62], 0.12

DSR= Dietary Species Richness, B =unstandardized beta regression coefficient, 95% CI = 95% confidence interval.
* = p-value <0.05
[1] adjusted for educational level (reference group = low educational level).
[2] adjusted for educational level and mean energy intake (kcal/day).
[3] adjusted for educational level, mean energy intake (kcal/day) and consumed amount of fruits/vegetables (grams).
°Included food groups: *fruits, vegetables, nuts and seeds, legumes, tubers, grains, meat, fish and dairy.*

Table S5. Sensitivity analysis of the association between Dietary Species Richness (DSR) and diet quality (DHD15-index score) in the Dutch National Food Consumption Survey 2012-2016 respondents (adults 19-79 years) adjusted for BMI instead of total kilocalories intake.

	**Model 1[1]**	**Model 2[2]**	**Model 3[3]**
Overall DSR°<br>*B [95%-CI], p-value*	1.22 [1.04-1.40]<br><0.001*	1.22 [1.04-1.40],<br><0.001*	-
DSR Fruit<br>*B [95%-CI], p-value*	3.77 [3.31-4.24],<br><0.001*	3.76 [3.29-4.22],<br><0.001*	1.68 [1.04-2.32]<br><0.001*
DSR Vegetables<br>*B [95%-CI], p-value*	0.89 [0.61-1.17],<br><0.001*	0.89 [0.61-1.17],<br><0.001*	0.22 [-0.06-0.50]<br>0.13

DSR= Dietary Species Richness, B =unstandardized beta regression coefficient, 95% CI = 95% confidence interval
* = p-value <0.05
[1] adjusted for sex and educational level (reference group = low educational level).
[2] adjusted for sex, educational level and Body Mass Index [kg/m²] (BMI).
[3] adjusted for sex, educational level, Body Mass Index [kg/m²] (BMI) and consumed amount of fruits/vegetables (grams).
° Included food groups: *fruits, vegetables, nuts and seeds, legumes, tubers, grains, meat, fish and dairy.*

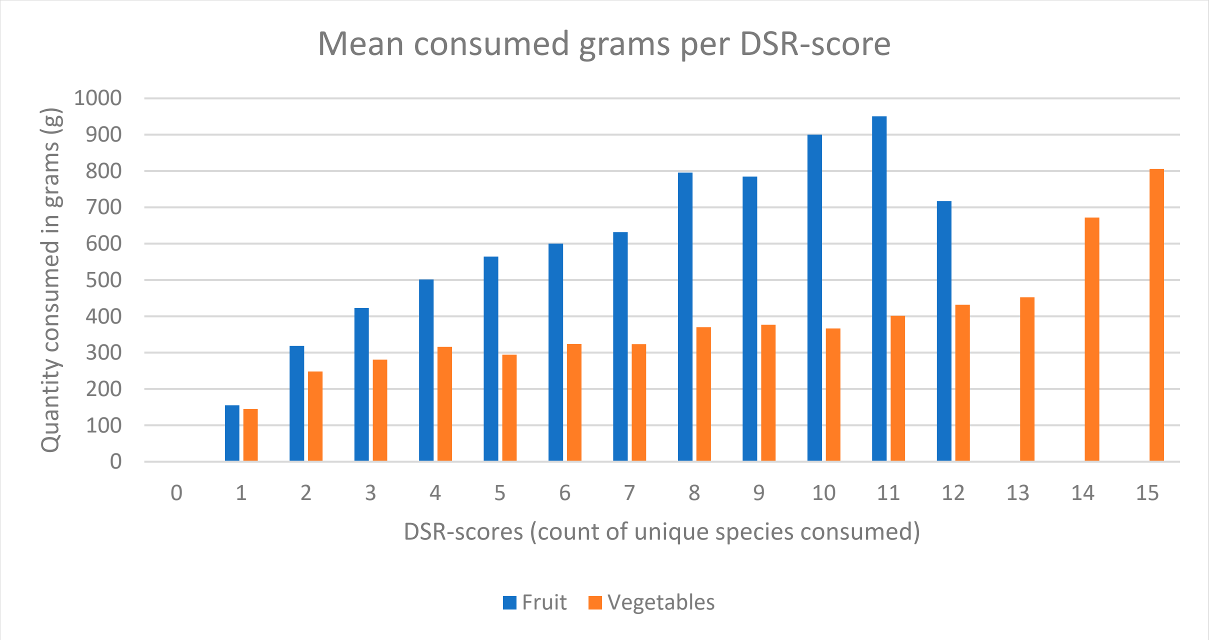

Figure S1. Graph of different Dietary Species Richness (DSR) scores and the quantity consumed in grams per DSR score in the Dutch National Food Consumption Survey 2012-2016 respondents (adults 19-79 years): DSR fruit and consumed quantity of fruit (blue), DSR vegetables and consumed quantity of vegetables (orange).

References:

1. Looman M, Feskens EJM, De Rijk M, Meijboom S, Biesbroek S, Temme EHM, et al. Development and evaluation of the Dutch Healthy Diet index 2015. Public Health Nutr. 2017 Sep 1;20(13):2289–99.
